# Impact of targeted drug administration and intermittent preventive treatment for forest goers using artesunate–pyronaridine to control malaria outbreaks in Cambodia

**DOI:** 10.1186/s41182-024-00607-2

**Published:** 2024-06-11

**Authors:** Dysoley Lek, Nguon Sokomar, Top Samphornarann, Jeanne Rideout, Saad El-Din Hassan, Tol Bunkea, Saing Sam Ath, Rothpisey Seng, John Hustedt, Thomas J. Peto, Jayme Hughes, Ke Kimmen, Khoy Dy, Bipin Adhikari

**Affiliations:** 1 National Center for Parasitology, Entomology, and Malaria Control, Phnom Penh, Cambodia; 2 School of Public Health, National Institute of Public Health, Phnom Penh, Cambodia; 3 Cambodia Malaria Elimination Project2, University Research Company Ltd., Phnom Penh, Cambodia; 4 FHI 360, Phnom Penh, Cambodia; 5 Mahidol-Oxford Tropical Medicine Research Unit, Faculty of Tropical Medicine, Mahidol University, Bangkok, Thailand; 6 Centre for Tropical Medicine and Global Health, Nuffield Department of Medicine, University of Oxford, Oxford, UK; 7 Clinton Health Access Initiative, Phnom Penh, Cambodia; 8 Provincial Health Department, Pursat, Cambodia

**Keywords:** Targeted drug administration, Intermittent preventive treatment, Outbreak response, forest goers, Artesunate–pyronaridine, Cambodia

## Abstract

**Introduction:**

The national malaria programme of Cambodia targets the rapid elimination of all human malaria by 2025. As clinical cases decline to near-elimination levels, a key strategy is the rapid identification of malaria outbreaks triggering effective action to interrupt local transmission. We report a comprehensive, multipronged management approach in response to a  2022 *Plasmodium falciparum* outbreak in Kravanh district, western Cambodia.

**Methods:**

The provincial health department of Pursat in conjunction with the Center for Parasitology, Entomology and Malaria Control (CNM) identified villages where transmission was occurring using clinical records, and initiated various interventions, including the distribution of insecticide-treated bed nets, running awareness campaigns, and implementing fever screening with targeted drug administration. Health stations were set up at forest entry points, and later, targeted drug administrations with artesunate–pyronaridine (Pyramax) and intermittent preventive treatment for forest goers (IPTf) were implemented in specific village foci. Data related to adherence and adverse events from IPTf and TDA were collected. The coverage rates of interventions were calculated, and local malaria infections were monitored.

**Results:**

A total of 942 individuals were screened through active fever surveillance in villages where IPTf and TDA were conducted. The study demonstrated high coverage and adherence rates in the targeted villages, with 92% (553/600) coverage in round one and 65% (387/600) in round two. Adherence rate was 99% (551/553) in round one and 98% (377/387) in round two. The study found that forest goers preferred taking Pyramax over repeated testing consistent with the coverage rates: 92% in round one compared to 65% in round two. All individuals reachable through health stations or mobile teams reported complete IPTf uptake. No severe adverse events were reported. Only six individuals reported mild adverse events, such as loss of energy, fever, abdominal pain, diarrhoea, and muscle aches. Two individuals attributed their symptoms to heavy alcohol intake following prophylaxis.

**Conclusions:**

The targeted malaria outbreak response demonstrated high acceptability, safety, and feasibility of the selected interventions. Malaria transmission was rapidly controlled using the available community resources. This experience suggests the effectiveness of the programmatic response for future outbreaks.

## Introduction

### Epidemiology of malaria in Cambodia

The reduction in malaria cases in Cambodia over the last two decades has been substantial, and the country is progressing towards malaria elimination at the sub-national level [1]. Malaria cases have reduced significantly from 106,905 cases in 2011 to only 4329 confirmed malaria cases in 2021 with no recorded deaths from malaria since 2018 [2]. In between July and September 2023, there were a total of 356 cases (2% *P. falciparum* and mixed cases, and 93% *P. vivax* cases), a 67% decrease compared to the same time period in 2022 [3]. If current trends continue, the National Malaria Control Programme of Cambodia (CNM) will succeed in eliminating all malaria by 2025. Historically, Cambodia has been an epicentre of antimalarial resistance [4]. The threat of spreading antimalarial resistance from western Cambodia has triggered substantial support from the global community for malaria elimination programmes and strategies in the Greater Mekong Subregion (GMS) [5, 6]. As the incidence of falciparum malaria is declining steadily in Cambodia, the slower decline in vivax malaria warrants species-specific strategies for the elimination [7]. Currently, more than 90% of malaria cases in Cambodia are caused by *P. vivax*. Occasional malaria outbreaks and relapsing vivax malaria warrant epidemiological surveillance, and the diligent implementation of containment strategies, including early diagnosis and appropriate treatment, to reach malaria elimination [8].

### Forest-acquired malaria is a major challenge for elimination in Cambodia

Much of the current malaria burden is concentrated among populations living in remote areas near the forest fringes or among forest workers who visit forests for their livelihood [9]. Although strategies to counteract forest malaria are difficult to design and require considerable resources to implement [9], several studies have reported on successful targeting of these populations, such as an antimalarial prophylaxis study conducted in 2020 in Siem Pang, north-eastern Cambodia [10]. The study showed that antimalarial prophylaxis can reduce the malaria burden among members of a highly mobile population. Implementation of such studies require teams of health workers to monitor and supervise malaria treatment and adherence to antimalarials [11]. An alternative is the establishment of the health service-structures conducting activities such as reaching out to high-risk populations during their forest visits, along with behavioural interventions such as the use of mosquito nets, and repellents. Nonetheless, the economic demands of such interventions can be significant and also require thorough evaluation.

### Global technical strategy and national malaria responses

The World Health Organization (WHO) has outlined Global Technical Strategies (GTS) for countries embarking on malaria elimination [12]. The national malaria Elimination Action Framework of Cambodia stresses access to malaria prevention, diagnosis, and treatment as part of universal health coverage. This involves targeting both parasites and vectors in transmission foci, as well as active case detection and case investigation as part of malaria surveillance and response programmes. In elimination settings, accelerated efforts include targeted drug administration in high-risk populations to reduce the infectious reservoir [13]. In addition, malaria surveillance which includes detecting outbreaks and assessing the impact of existing interventions is a key strategy in the last mile phase of malaria elimination.

The national malaria programme of Cambodia (CNM) has strengthened the ongoing surveillance programmes and recently responded to a focal malaria outbreak in Kravanh district by using a multi-pronged approach that included (1) insecticide-treated mosquito nets (ITN) and insect repellent distribution; (2) community engagement and social and behaviour change (SBC) activities; (3) daily active fever screening followed by targeted drug administration (TDA) with Pyramax (artesunate + pyronaridine) and intermittent preventive treatment for forest goers (IPTf) in three villages at forest entry points with mobile teams. The main objective of this study was to describe and evaluate the implementation of the national response to a focal malaria outbreak that occurred in Kravanh district of Cambodia during 2021 and 2022.

## Materials and methods

### Background on epidemiological response

The Phnom Kravanh (PKV) operational district (OD) in Pursat province includes two administrative districts (Phnom Kravanh and Veal Veang) and 10 health facilities including one referral hospital. A total of 81 village malaria workers (VMWs) and mobile malaria workers (MMWs) were active at community level during the study period.

**Fig. 1:**
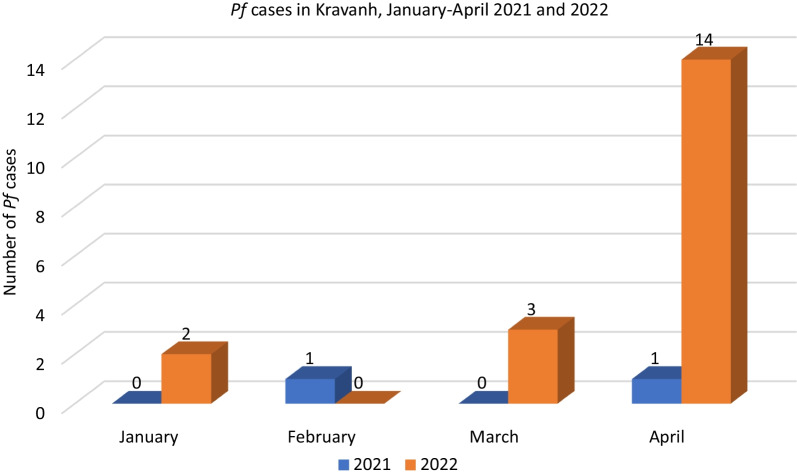
Number of *Plasmodium falciparum* cases in Kravanh district between January and April 2021 and 2022

During 2020 and 2021, most *Plasmodium falciparum *(*Pf*) cases in the district occurred during the rainy season. Only two cases were reported between October 2020 and June 2021. In the first quarter of 2022, two cases were reported in January, none in February and three in March. In April, a total of 14 *Pf* cases were reported, compared to only one case in 2021 (Fig. 1).

**Fig. 2:**
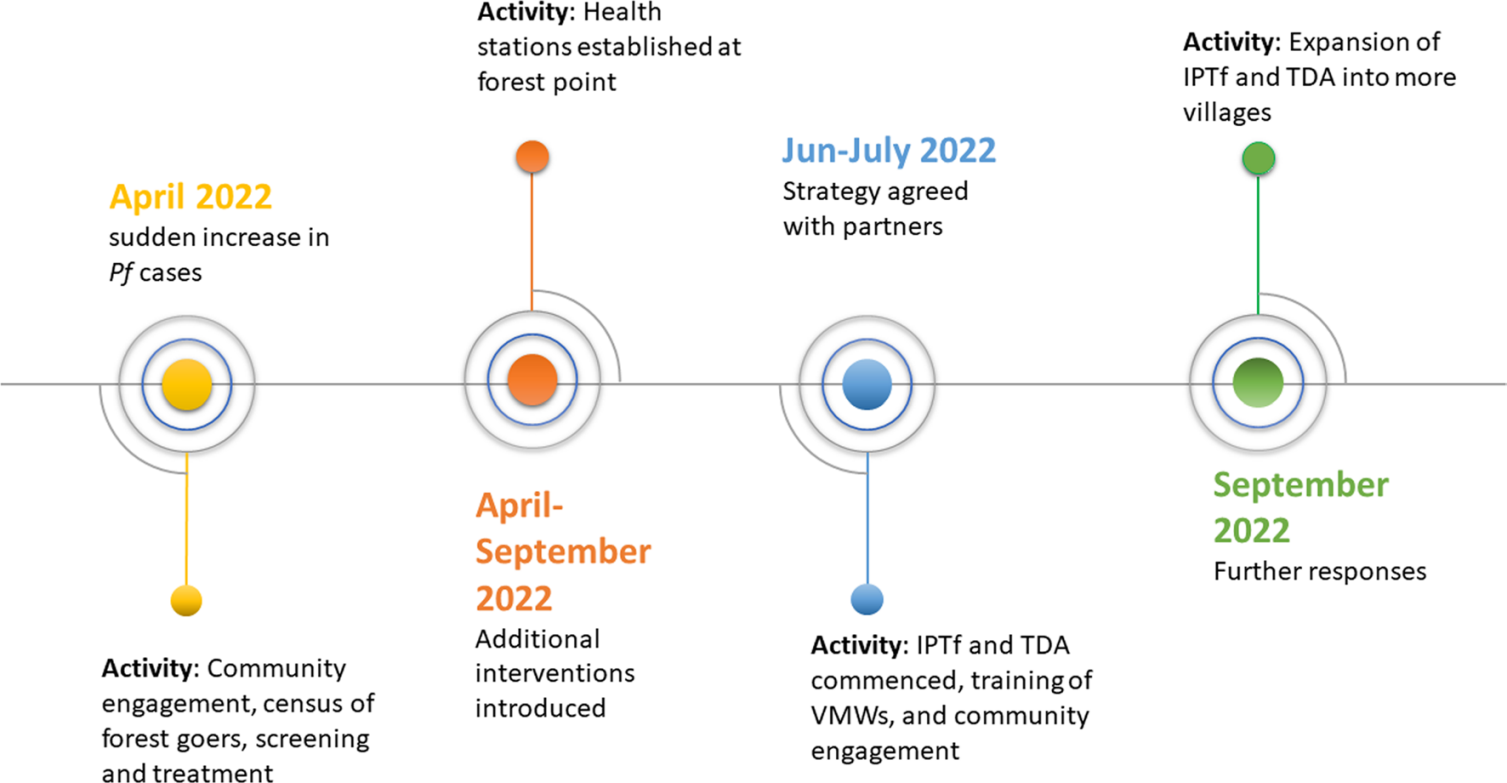
Timeline showing programmatic responses to outbreak. Main programmatic responses included Intermittent Preventive Treatment for Forest goers (IPTf) and Targeted Drug Administration (TDA)

On April 24th, a sudden increase in *Pf* cases was reported, and the OD introduced a package of interventions (Fig. 2). This started with a census of active forest goers in the village and was followed by screening for and treatment of malaria in five villages where an increase in *Pf* cases was reported (Veal Vong, Mol Rokat, Veal, Ou Preal and Prek II). Subsequently, the following interventions were introduced: (1) ITN and repellent distribution; (2) public service announcements and social and behaviour change activities (community engagement); and (3) daily active fever screening.

Stationary points for health services (health stations) at forest entry and exit points were established first in April at Sre Bon and Pich Sla locations. Based on epidemiological data, these were combined soon thereafter into a single health station, further into the forest, at Khnor Rolou. The health stations have been operational 24 h a day starting from May 23rd, 2022. A second health station (Kampong Touk) was established on July 1st, and a third health station (Ou Chrov) on September 3rd, 2022.

Following the establishment of health stations, a meeting was held, led by CNM with its partners (Global Fund, WHO and other stakeholders) on June 13th, 2022, who agreed to implement TDA with Pyramax in one village (Cham Ka Phnom—a sub-village of Kset Borey) with an identified malaria case. During the meeting it was agreed to implement intermittent preventive treatment for forest goers (IPTf) in three villages (Mol Rokat, Veal Vong and Veal) which reported *Pf* cases for two successive months.

On July 5th, 2022, preparation for IPTf and TDA began which entailed training local authorities and health teams, community engagement (CE) sessions, a population census, and active fever screening. An additional 19 VMWs were assigned to work alongside the original six VMWs. The added VMWs comprised both newly recruited VMWs as well as volunteers reassigned from other areas where the demand for their service was lower. The village census conducted at the start of IPTf/TDA implementation was used to identify the target population in the selected four villages. Based on known propensity, that is likelihood to visit forest, male forest goers aged 15–49 were identified by VMWs and the village chief. The definition of ‘forest goer’ was flexible and included individuals who had travelled to the forest in the past but not necessarily during the assessment period. The target population remained the same throughout the TDA/IPTf implementation over several rounds.

**Fig. 3:**
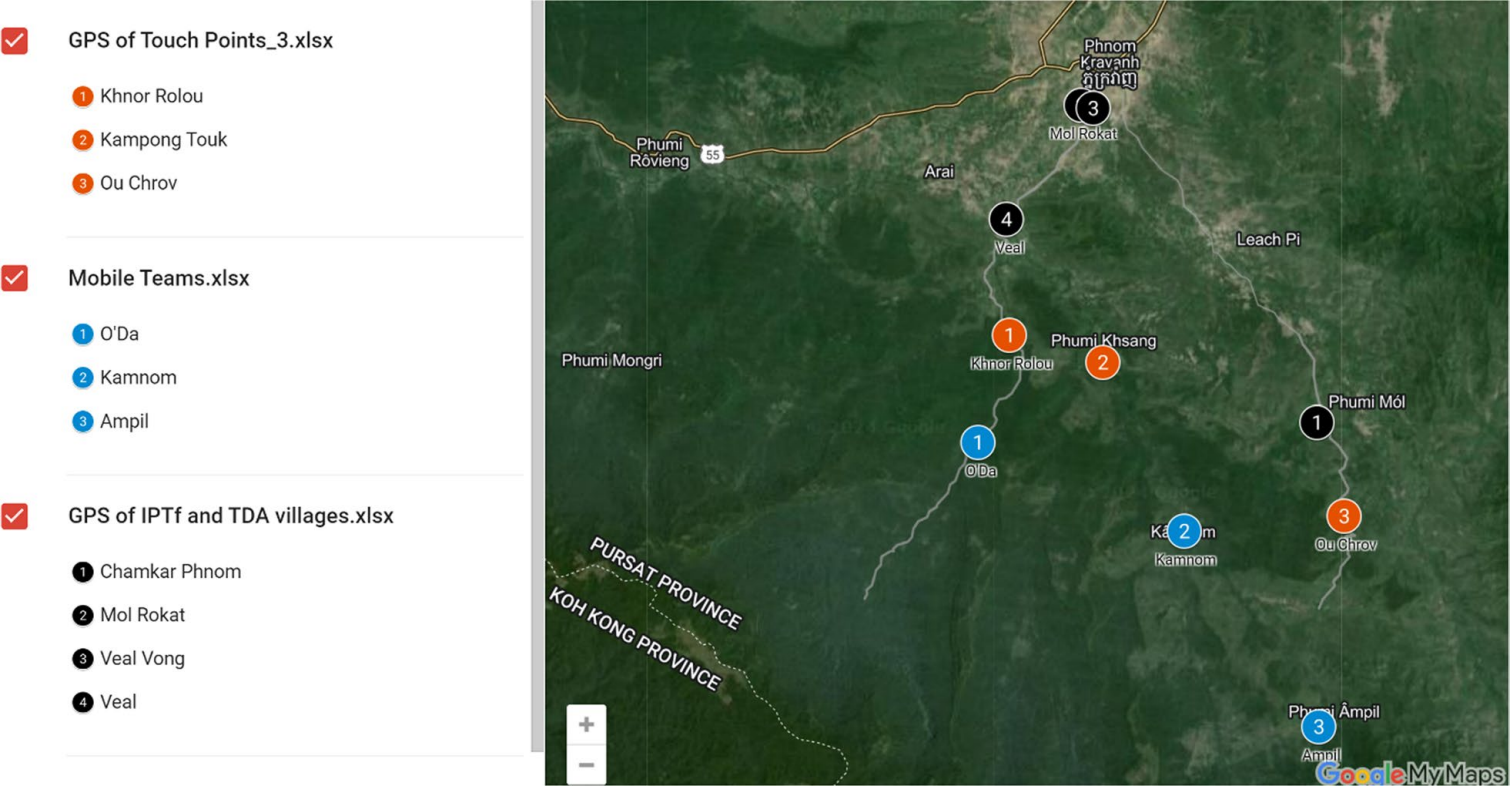
Location of Intermittent Preventive Treatment for Forest goers (IPTf) and Targeted Drug Administration (TDA) villages, mobile teams, and health stations

Following implementation of IPTf in the first three villages, the same intervention was added at the newly established health station of O’Chrov with the help of mobile teams on September 3rd, 2022 (Fig. 3). All men aged 15–49 who tested negative by the mobile teams and at the O’Chrov health station were given IPTf. Following the test, the first dose of Pyramax was administered using a directly observed treatment (DOT) strategy, and forest goers were provided two doses for next two consecutive days. The teams then called the forest goers to verify adherence and detect adverse events. Contacting forest goers was challenging. Only 19% of forest-goers were reachable by phone.

During an urgent meeting convened by CNM in early September 2022 to discuss the malaria situation in Pursat, it was agreed to conduct IPTf in eight additional villages following reports of increasing number of *Pf* cases between January and September 2022: Veal and Veal Ampos, Mol Rokat, Veal Vong, Kset Borey, Preak Pi, Ou Preal, Kol Totueng and Paen. Between October and November, seven *Pf* cases were recorded in Kravanh with five in the proposed IPTf villages: Kset Borey (*n* = 2), Ou Preal (*n* = 1), Kol Totueng (*n* = 1) and Paen (*n* = 1) and two villages where no interventions had been conducted: Cheu (*n* = 1) and Mol Chas (*n* = 1). Ultimately, TDA was conducted in one village (Cham Ka Phnom, a sub-village of Kset Borey). IPTf was conducted in Mol Rokat, Veal Vong, and Veal villages, at O’Chrov health stations and through mobile teams.

### Data collection

Each individual residing in the target villages had their name, age, weight, number of tablets given, date of DOT for rounds one, two and three. Contact information were recorded on a case record form at the site of administration and were collected by a Malaria Elimination Team (MEL). The MEL team recorded this information into an Excel file, which was subsequently verified by the cluster lead and was used for analysis. All individuals seen at the health station or reached by mobile teams were called at the contact number they provided to verify whether they had taken the second and third doses, and if they had any Adverse Events (AEs).

Field teams met with the VMWs/MMWs to explore if anyone who participated in TDA or IPTf tested positive for malaria in the village. Data from the MIS (national malaria information system maintained in web: https://mis.cnm.gov.kh/Dashboard) was reviewed to assess if any of the individuals given TDA or IPTf tested positive outside the village. In addition to routine detection of malaria cases at the points of care, all three IPTf targeted villages and the TDA sub-village were undergoing active fever screening during the study period.

### Analysis

Coverage was calculated by dividing the number that received TDA/IPTf by the total number of eligible males 15–49 who travelled to the forest. The percentage of individuals considered ‘adherent’ was estimated by the following formula: (number of individuals who completed the full required course/number of individuals who took at least one dose) multiplied by 100. As coverage and adherence were not directly observed by the team for the full three-day doses at O’Chrov health station and by mobile teams, these were not included in the coverage/adherence results. The total number of individuals who received IPTf at the health station and by mobile teams was 14% of the participants included in round one and 8% of the participants included in round two.

## Results

The data showed combined coverage (of both TDA and IPTf) of 92% in round one and 65% in round two, and adherence to the intervention was 99% in round one and 92% in round two in the four target villages (Table 1). All the individuals who could be contacted took the complete dose. No Severe Adverse Events (SAEs) were reported, and only six individuals reported AEs, which included loss of energy (two individuals), fever (four individuals), and stomach-ache, diarrhoea, and muscle aches (one individual). Two individuals reported drinking alcohol heavily following the prophylaxis and felt that it might have contributed to their symptoms. None reported symptoms that lasted more than two days.

**Table 1:** Results from Targeted Drug Administration (TDA) and Intermittent Preventive Treatment for Forest goers (IPTf), July–October 2022

				Round 1		Round 2			
Location	Pop	Pop (Male 15–49)	Target Pop (Travel to forest)	Treated > 1 dose (Coverage)	Treated-3 doses (Adherence)	Treated > 1 dose (Coverage	Treated-3 doses (Adherence)	SAE	AEs
Cham Ka Phnom (TDA)	224	60	60	60/60 (100%)	59/60 (98%)	42/60 (70%)	41/42 (98%)	0	2
Mol Rokat (IPTf)	1119	222	87	81/87 (93%)	81/81 (100%)	71/87 (82%)	63/71 (89%)	0	0
Veal Vong (IPTf)	1791	437	270	239/270 (89%)	238/239 (99%)	149/270 (55%)	149/149 (100%)	0	0
Veal (IPTf)	1390	335	183	173/183 (95%)	173/173 (100%)	125/183 (68%)	124/125 (99%)	0	0
O’Chrov (IPTf)	N/A	N/A	N/A	23	–	–	–	0	2
Mobile Teams (IPTf)	N/A	N/A	N/A	65	–	21	–	0	2
Total	4524	737	600	641 [553 (92%) *]	551 (99%)	408 [387 (65%)*]	377 (98%)	0	6

*The total number of doses was summed and provided in the total column, while the total number of doses provided to individuals in villages are in brackets

Coverage was determined by dividing the total number of doses that were given in IPTf and TDA villages which had participating populations divided by the total target populations. The coverage calculations did not include those individuals reached at O’Chrov or by mobile teams.

**Fig. 4:**
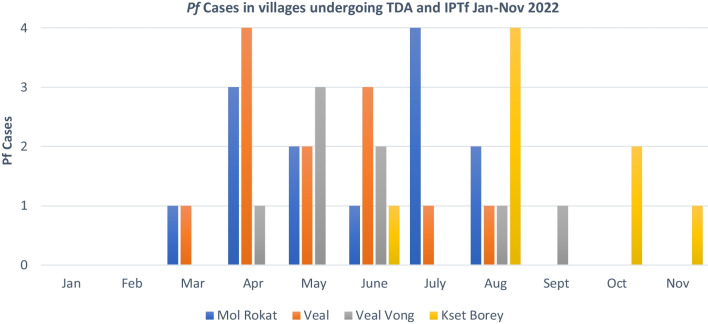
*Plasmodium falciparum* cases in villages eligible for Targeted Drug Administration (TDA) and Intermittent Preventive Treatment for Forest goers (IPTf) in Phnom Kravanh, Jan–Nov 2022

The MIS database, and VMW/MMW logbooks did not show any reported malaria cases among individuals who took prophylaxis using Pyramax in PKV. The *Pf* cases from January to November 2022 originating from the TDA and IPTf targeted villages are displayed in Fig. 4. All *Pf* cases in PKV from January to November are reported in Appendix 1. All cases except one (the case from Cham ka Phnom sub-village in Kset Borey) had recent travel outside the village into the forest. The diagnosis location and potential exposure location (where they travelled in the last two weeks) of all cases from April–November 2022 were tracked through Global Positioning System (GPS). Nine hundred and forty-two individuals were screened through active fever screening in the IPTf and TDA villages. Based on our screening at point of entry with the forest goers, the preference to take IPTf with Pyramax was high (round one: 553/600; 92% versus round two: 387/600; 65%) over being tested repeatedly. We observed growing opposition to testing and avoidance of test points over the study period.

## Discussion

As Cambodia prepares for malaria elimination, surveillance and active detection of malaria cases in high-risk areas and populations becomes the central strategy [13–15]. CNM included this strategy in its elimination action framework 2021–2025 that includes (1) early case detection and treatment; (2) intensification of focal interventions in endemic locations and among high-risk populations (e.g., mobile and migrant population, and forest goers) and (3) investigation of 100% of cases, including clearing the cases, and documenting them [16].

The study offers a pragmatic lesson for future programmatic roll out of the package of interventions, particularly in malaria endemic villages and among specific populations such as forest goers. These results are important for CNM as they are the first to demonstrate the feasibility of malaria chemoprevention response strategies in an elimination setting. Provided adequate preparations are in place, programmatic roll out can be conducted with the resources available within the operational budget of the health system utilizing community-based network of VMWs and MMWs as opposed to conducting large scale research studies that require additional resources [17]. The fact that the study was conducted in the malaria endemic villages by the community based VMWs and MMWs also demonstrates the value of these locally available resources and the feasibility of their effectiveness during outbreaks [18]. Community based implementation of interventions can be a feasible option when accompanied by adequate community engagement [11]. Community engagement has been established to support and facilitate the implementation of large scale, community wide public health interventions [19–23]. Utilizing local resources including community members can foster the trust and relationship between the program implementers and the community members [24, 25]. The wider implications of such operational studies bear the potential for sustainability and independence in research and reporting which could be relevant for other malaria endemic countries/settings. The findings from this outbreak response suggest the utility and effectiveness of the interventions employed by the study.

Using passive case detection, no malaria case was reported among the group that received the intervention. However, no additional samples were collected from individuals who received prophylaxis. This is due to the nature of programmatic implementation as opposed to efficacy or effectiveness studies [26]. The intervention reported in this study did not aim to evaluate the effectiveness of TDA or IPTf. Operational research can assess effective targeting, feasibility, and optimization, bridging the gap between traditional methods and the more practical side of service and program implementation [27–30]. In this study, adherence to the first round exceeded 90% aligning with mass antimalarial administration interventions carried out in various countries within GMS [31–34]. A recent study conducted in Stung Treng, north-eastern Cambodia found high adherence to and effectiveness of antimalarial prophylaxis among forest goers successfully clearing the malaria parasites [10]. No severe adverse events were associated with the interventions, and only a few minor adverse events related to  the antimalarials were reported, consistent with prior mass antimalarial administration interventions in the GMS [31–34]. As part of the programmatic intervention, no detailed explorations, apart from brief clinical history, were conducted on their existing health conditions and potential risk factors that could have predisposed or accentuated these adverse events.

Around one third of individuals (*n* = 166) who received prophylaxis in round one did not receive it in round two. The forest goers who refused to take a second dose reported that they had stopped their visits to the forest. It was difficult to identify frequent forest goers, mainly because of the nature of work [35]. Forest workers are recognized to be a vulnerable population in terms of the malaria risk, and delayed treatment seeking behaviour including uptake of community-based health services offered by VMWs and MMWs [36–38]. The categorization of these forest goers was based on the assessment by the village administration. The coverage could have been higher if those who claimed to be forest goers but did not actually visit forests were excluded. Changes in human behaviour, such as banning visits to the forest, and reporting biases are often challenges associated with ‘at risk populations’ echoing previous forest prophylactic study conducted in north-eastern Cambodia [10, 11]. This operational study underscores the need to strengthen community-based resources through engagement when targeting high risk populations such as mobile populations, migrants, forest goers, and swidden cultivators [10, 11, 39].

## Conclusions

The study showed that malaria outbreaks can be managed using existing resources available in the health system, namely VMWs and MMWs. The programmatic roll out of the IPTf and TDA are an example of how focal responses to malaria outbreaks are conducted in settings embarking on malaria elimination. While their effectiveness cannot be assessed with the same methodological rigor as effectiveness studies, the observed operational feasibility and absence of malaria incidence among those receiving the intervention suggest a potential benefit of such interventions.

## Data Availability

All data are freely available upon request to CNM and CMEP2. The data are also available freely at CNM’s electronic database maintained at https://mis.cnm.gov.kh/Dashboard.

## Appendix

*Plasmodium falciparum* cases in Kravanh district of Pursat province, January to November 2022

**Table:**

Village name	JAN	FEB	MAR	APR	MAY	JUN	JUL	AUG	SEP	OCT	NOV	Total
Mol Rokat	0	0	1	3	2	1	4	2	0	0	0	**13**
Veal	0	0	1	4	2	3	1	1	0	0	0	**12**
Kol Totueng	2	0	0	0	0	1	0	2	2	1	0	**8**
Kset Borey	0	0	0			1	0	4		2	1	**8**
Veal Vong	0	0	0	1	3	2	0	1	1	0	0	**8**
Veal Ampos	0	0	0	0	1	7	0	0		0	0	**8**
Preak Pi	0	0	0	2	1	0	0	0	4	0	0	**7**
Paen	0	0	0	0	1	0	0	2	1	1	0	**5**
Srae Popeay	0	0	0	0	0	0	0	2	2	0	0	**4**
Prongil	0	0	0	0	0	0	0	0	3	0	0	**3**
Ou Preal	0	0	0	2	0	0	0	0	0	1	0	**3**
Baoh Puoy	0	0	0	0	0	0	0	1	1	0	0	**2**
Sbov Rik	0	0	0	0	0	1	0	0	1	0	0	**2**
Taing Yar	0	0	0	0	**2**		0	0	0	0	0	**2**
Samraong Yea	0	0	0	0		2	0	0	0	0	0	**2**
Ou Heng	0	0	0	0		1	0	1	0	0	0	**2**
Prey Smach	0	0	0	0	1	0	0	0	0	0	0	**1**
Santreae	0	0	0	0		0	0	0	1	0	0	**1**
Krouch Chhmar	0	0	0	0	1	0	0	0	0	0	0	**1**
Prey Khlong	0	0	0	0	0	1	0	0	0	0	0	**1**
Mul Chas	0	0	0	0	0	0	0	0	0	0	1	**1**
Mul Chas Dam Nak Thlok	0	0	0	0	0	0	0	1	0	0	0	**1**
Veal Vong Khsang Kanom	0	0	0	1	0	0	0	0	0	0	0	**1**
Doun Neak	0	0	0	1	0	0	0	0	0	0	0	**1**
Pech Ban	0	0	0	0	0	0	0	1	0	0	0	**1**
Angkrong	0	0	1	0	0	0	0	0	0	0	0	**1**
Preaek Muoy	0	0	0	0	0	0	0	0	1	0	0	**1**
Samraong Muoy	0	0	0	0	1	0	0	0	0	0	0	**1**
**Total**	**2**	**0**	**3**	**14**	**15**	**20**	**5**	**18**	**17**	**5**	**2**	**101**

*Note these data are by date of diagnosis rather than date of report

## Abbreviations

AEs: Adverse events
COVID-19: Corona Virus Infectious Diseases-2019
CNM: National Center for Parasitology, Entomology and Malaria Control
CMEP2: Cambodia Malaria Elimination Project 2
DOT: Directly observed treatment
GMS: Greater Mekong Subregion
GTS: Global technical strategy
MEL: Malaria elimination team
MMWs: Mobile malaria workers
IPTf: Intermittent preventive treatment for forest goers
PMI: Presidents’ malaria initiative
TDA: Targeted Drug Administration
VMWs: Village malaria workers
SAEs: Severe adverse events
SBC: Social and behaviour change
WHO: World Health Organization
ITN: Insecticide treated nets
USAID: United States Agency for International Development
